# SurvCurv database and online survival analysis platform update

**DOI:** 10.1093/bioinformatics/btv463

**Published:** 2015-08-06

**Authors:** Matthias Ziehm, Dobril K. Ivanov, Aditi Bhat, Linda Partridge, Janet M. Thornton

**Affiliations:** 1 ^1^European Molecular Biology Laboratory, European Bioinformatics Institute (EMBL-EBI), Wellcome Trust Genome Campus, Hinxton, Cambridge CB10 1SD, UK,; 2 ^2^Department of Genetics, Evolution and Environment, The Institute of Healthy Ageing, University College London, London WC1E 6BT, UK and; 3 ^3^Max Planck Institute for Biology of Ageing, 50931 Cologne, Germany

## Abstract

**Summary:** Understanding the biology of ageing is an important and complex challenge. Survival experiments are one of the primary approaches for measuring changes in ageing. Here, we present a major update to SurvCurv, a database and online resource for survival data in animals. As well as a substantial increase in data and additions to existing graphical and statistical survival analysis features, SurvCurv now includes extended mathematical mortality modelling functions and survival density plots for more advanced representation of groups of survival cohorts.

**Availability and implementation:** The database is freely available at https://www.ebi.ac.uk/thornton-srv/databases/SurvCurv/. All data are published under the Creative Commons Attribution License.

**Contact:** matthias.ziehm@ebi.ac.uk

**Supplementary information:** Supplementary data are available at *Bioinformatics* online.

## 1 Introduction

The biological phenomenon of ageing has been a long standing area of research (Grignolio and Franceschi, 2012). Despite identification of many gene knock-outs, drug administration and environmental regimes that can extend lifespan of model organisms under laboratory conditions, many questions remain unresolved (Guarente *et al.*, 2008). A key experimental approach in research on ageing is to use longevity survival experiments, which usually characterize differences with respect to ageing between populations when other causes of death, such as predators, food competition and pathogens, are excluded. Until recently, numerical data from animal survival experiments were usually not shared, and the data were analysed only in the original study, hampering method development, re-analysis of existing data by new methods, meta-analyses combining data and systems biology approaches.

We have created the SurvCurv database and online analysis platform for animal survival data (Ziehm and Thornton, 2013). Here, we present a major SurvCurv update, comprising improvements and new analysis features as well as increased data content.

## 2 Description

SurvCurv is a database and analysis platform accessible through its user-friendly web interface at https://www.ebi.ac.uk/thornton-srv/databases/SurvCurv/. The main website gives access to the database through a search box, querying the manually curated annotations of experimental conditions and treatments. Annotations use existing databases and ontologies, where possible, and advanced searches using specific database fields are available. All data from the database can be analysed online and additional user data can be analysed alone or in combination with database content. Various data formats for file uploads are supported. The database has more than doubled its data content since the original release and currently contains survival records for more than 300  000 individuals from various organisms including 160  000 *Caenorhabditis elegans*, 130  000 *Drosophila* and for the first time a large set of 12  000 mice. Survival records belong to a total of 4226 cohorts from 60 publications (see Supplementary Material) and include survival under various conditions such as different temperatures, dietary conditions, genetic mutations and drug administration regimes.

### 2.1 Analysis features

The SurvCurv analysis features include basic Kaplan–Meier survival plots showing the percentage of the population alive over time. Additional representations include incidence plots, showing the distribution of deaths, and log mortality plots, showing the instantaneous log-risk of dying. Plots are available in different graphics formats including PNG, SVG and PDF.

Statistical differences in survival between cohorts can be tested using the log-rank test or generalized Wilcoxon test. The Wang–Allison test (Wang *et al.*, 2004) for difference in ‘maximal’ survival and Fisher’s exact test (Fisher, 1934) for difference at a user-defined time point can be applied, as can Cox proportional hazards analysis (Cox, 1972) with Efron approximation for ties. Cox analysis features include interaction terms, an automatically conducted test of the proportional hazards assumption and diagnostic plots showing the scaled Schoenfeld residuals (see FAQ online and Grambsch and Therneau, 1994 for more information).

All public cohorts are published under the Creative Commons Attribution License (http://creativecommons.org/licenses/by/3.0/) and can be directly downloaded individually in various file formats. Whole database MySQL dumps are provided upon request.

### 2.2 New features

**Fig. 1.**
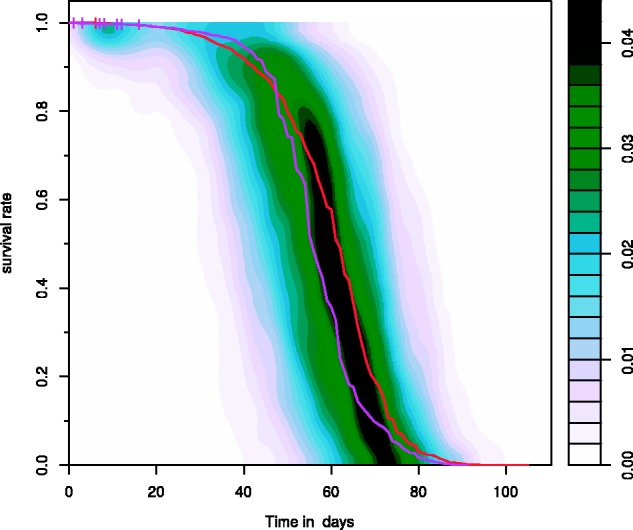
Example of a survival density plot showing the variation of female survival of 290 *Drosophila* Genetic Reference Panel (DGRP) lines overlaid with the overall survival of female controls of *Drosophila* strains wDah (red) and w1118 (purple) from other experiments

In addition to the large increase in data, a major new feature is the ability to generate survival density plots. This novel visualization shows the distribution of a group of survival curves as a two-dimensional density, which can be combined with survival plots of individual cohorts superimposed on top (see Fig. 1). Survival density plots are composed of horizontal density estimates, which make it highly sensitive for differences in the decreasing section of survival curves usually of interest, while the typical initial horizontal stretch is underestimated.

Previously, mortality models were only calculated once the data were inserted in the database. Now, the full set of six common mathematical mortality models (Exponential, Weibull, Gompertz, Gompertz-Makeham, Logistic and Logistic-Makeham model) can be directly calculated online for data uploaded by the user for analysis and user-defined meta-cohorts. In addition, all data from the database or user-files can now be analysed by pair-wise joint mortality modelling and detection of significant model improvement by separately fitted single parameters, i.e. detection of significantly different optimal model parameters. This analysis determines if two survival cohorts differ with respect to a specific mortality model factor, e.g. baseline hazard rate, rather than in general or at a specific time, thereby markedly extending the diagnostic options.

Moreover, the new release of SurvCurv contains various additional features and improvements, including colouring schemes and time units for plotting and plot output as a list of *x* and *y* values for plotting in external programs for customized graphics.

### 2.3 Implementation

The web interface is implemented in PHP on an Apache webserver using secure https for all connections. All data are stored in a MySQL database. Single cohort mortality models and descriptive statistics are pre-calculated and cached for quick retrieval.

Genes and alleles are annotated using organism-specific databases such as FlyBase (Dos Santos *et al.*, 2015), WormBase (Harris *et al.*, 2014) or MGI (Eppig *et al.*, 2015). Species are referenced through NCBI Taxonomy, compounds annotated using ChEBI identifiers (Hastings *et al.*, 2013), units specified using Unit Ontology and publications referenced through PubMed ID or DOI, where possible.

Visualization and survival analysis functions are realized via server-side R scripts using the survival package (Therneau, 2015) and the Survomatic package (Bokov and Gelfond, 2010), which includes an R port of WinModest (Pletcher, 1999). We implemented the Wang–Allison test in R. Survival density plots were implemented using independent 1D density estimates for each 2-percentage survival band across all cohorts and times using a Gaussian kernel with a smoothing bandwidth computed by Silverman’s rule of thumb (Silverman, 1986, eq 3.31). The results are smoothed using directly neighbouring survival bands.

## Supplementary Material

Supplementary Data
