# Supplementary material for: SurvCurv database and online survival analysis platform update
Source: Bioinformatics. 2015 Aug 6;31(23):3878–80. doi: 10.1093/bioinformatics/btv463 (PMC4653391; doi:10.1093/bioinformatics/btv463)
Supplement: Supplementary Data [file supp_31_23_3878__index.html]

SurvCurv database and online survival analysis platform update — SurvCurv database and online survival analysis platform update — Supplementary Data 

# SurvCurv database and online survival analysis platform update

## Supplementary Data

files

- Supplementary Data - pdf file
