# Supplementary material for: SurvCurv database and online survival analysis platform update
Source: Bioinformatics. 2015 Aug 6;31(23):3878–80. doi: 10.1093/bioinformatics/btv463 (PMC4653391; doi:10.1093/bioinformatics/btv463)
Supplement: Supplementary Data [file supp_btv463_SupplementListOfRefences.pdf]

References of all publications currently available in SurvCurv

- Alic, N., *et al.* (2012) Detrimental effects of RNAi: a cautionary note on its use in Drosophila ageing studies, *PLoS One*, **7**, e45367.
- Alic, N., *et al.* (2011) Lifespan extension by increased expression of the Drosophila homologue of the IGFBP7 tumour suppressor, *Aging Cell*, **10**, 137-147.
- Ayyadevara, S., *et al.* (2008) Remarkable longevity and stress resistance of nematode PI3K-null mutants, *Aging Cell*, **7**, 13-22.
- Barnes, A.I., *et al.* (2008) Feeding, fecundity and lifespan in female Drosophila melanogaster, *Proc Biol Sci*, **275**, 1675-1683.
- Bjedov, I., *et al.* (2010) Mechanisms of life span extension by rapamycin in the fruit fly Drosophila melanogaster, *Cell Metab*, **11**, 35-46.
- Broughton, S., *et al.* (2008) Reduction of DILP2 in Drosophila triages a metabolic phenotype from lifespan revealing redundancy and compensation among DILPs, *PLoS One*, **3**, e3721-e3721.
- Broughton, S.J., *et al.* (2010) DILP-producing median neurosecretory cells in the Drosophila brain mediate the response of lifespan to nutrition, *Aging Cell*, **9**, 336-346.
- Burger, J.M., Buechel, S.D. and Kawecki, T.J. (2010) Dietary restriction affects lifespan but not cognitive aging in Drosophila melanogaster, *Aging Cell*, **9**, 327-335.
- Burger, J.M.S., *et al.* (2007) The functional costs and benefits of dietary restriction in Drosophila, *Aging Cell*, **6**, 63-71.
- Bushey, D., *et al.* (2010) Sleep, aging, and lifespan in Drosophila, *BMC Neurosci*, **11**, 56.
- Bushey, D., Tononi, G. and Cirelli, C. (2009) The Drosophila fragile X mental retardation gene regulates sleep need, *J Neurosci*, **29**, 1948-1961.
- Carey, J.R., *et al.* (2008) Longevity-fertility trade-offs in the tephritid fruit fly, Anastrepha ludens, across dietary-restriction gradients, *Aging Cell*, **7**, 470-477.
- Chapman, T., *et al.* (2001) The Acp26Aa seminal fluid protein is a modulator of early egg hatchability in Drosophila melanogaster, *Proc Biol Sci*, **268**, 1647-1654.
- Chapman, T., Hutchings, J. and Partridge, L. (1993) No reduction in the cost of mating for Drosophila melanogaster females mating with spermless males, *Proc Biol Sci*, **253**, 211-217.
- Chapman, T., *et al.* (1995) Cost of mating in Drosophila melanogaster females is mediated by male accessory gland products, *Nature*, **373**, 241-244.
- Chapman, T., *et al.* (1998) Interactions of mating, egg production and death rates in females of the Mediterranean fruit fly, Ceratitis capitata, *Proc Biol Sci*, **265**, 1879-1894.
- Clancy, D.J. (2008) Variation in mitochondrial genotype has substantial lifespan effects which may be modulated by nuclear background, *Aging Cell*, **7**, 795-804.
- Defays, R., *et al.* (2011) Quantitative trait loci for longevity in heat-stressed Drosophila melanogaster, *Exp Gerontol*, **46**, 819-826.
- Fisher, A.L. and Lithgow, G.J. (2006) The nuclear hormone receptor DAF-12 has opposing effects on Caenorhabditis elegans lifespan and regulates genes repressed in multiple long-lived worms, *Aging Cell*, **5**, 127-138.
- Gharbi, H., *et al.* (2013) Loss of the Birt-Hogg-Dube gene product folliculin induces longevity in a hypoxia-inducible factor-dependent manner, *Aging Cell*, **12**, 593-603.
- Giannakou, M.E., *et al.* (2007) Dynamics of the action of dFOXO on adult mortality in Drosophila, *Aging Cell*, **6**, 429-438.

- Giannakou, M.E., *et al.* (2004) Long-lived Drosophila with overexpressed dFOXO in adult fat body, *Science*, **305**, 361-361.
- Goddeeris, M.M., *et al.* (2003) Delayed behavioural aging and altered mortality in Drosophila beta integrin mutants, *Aging Cell*, **2**, 257-264.
- Gomez, A., *et al.* (2014) Lifelong treatment with atenolol decreases membrane fatty acid unsaturation and oxidative stress in heart and skeletal muscle mitochondria and improves immunity and behavior, without changing mice longevity, *Aging Cell*, **13**, 551-560.
- Grönke, S., *et al.* (2010) Molecular evolution and functional characterization of Drosophila insulin-like peptides, *PLoS Genet*, **6**, e1000857-e1000857.
- Harrison, D.E., *et al.* (2014) Acarbose, 17-alpha-estradiol, and nordihydroguaiaretic acid extend mouse lifespan preferentially in males, *Aging Cell*, **13**, 273-282.
- Harrison, D.E., *et al.* (2009) Rapamycin fed late in life extends lifespan in genetically heterogeneous mice, *Nature*, **460**, 392-395.
- Ikeya, T., *et al.* (2009) The endosymbiont Wolbachia increases insulin/IGF-like signalling in Drosophila, *Proc Biol Sci*, **276**, 3799-3807.
- Iqbal, A., *et al.* (2009) Chemical changes in aging Drosophila melanogaster, *Age (Dordr)*, **31**, 343-351.
- Jacobson, J., *et al.* (2010) Biomarkers of aging in Drosophila, *Aging Cell*, **9**, 466-477.
- Kerr, F., *et al.* (2011) Dietary restriction delays aging, but not neuronal dysfunction, in Drosophila models of Alzheimer's disease, *Neurobiol Aging*, **32**, 1977-1989.
- Liao, C.-Y., *et al.* (2010) Genetic variation in the murine lifespan response to dietary restriction: from life extension to life shortening, *Aging Cell*, **9**, 92-95.
- Masse, I., *et al.* (2008) A novel role for the SMG-1 kinase in lifespan and oxidative stress resistance in Caenorhabditis elegans, *PLoS One*, **3**, e3354-e3354.
- McCulloch, D. and Gems, D. (2003) Body size, insulin/IGF signaling and aging in the nematode Caenorhabditis elegans, *Exp Gerontol*, **38**, 129-136.
- McCulloch, D. and Gems, D. (2003) Evolution of male longevity bias in nematodes, *Aging Cell*, **2**, 165-173.
- Melvin, R.G. and Ballard, J.W.O. (2006) Intraspecific variation in survival and mitochondrial oxidative phosphorylation in wild-caught Drosophila simulans, *Aging Cell*, **5**, 225-233.
- Miller, R.A., *et al.* (2011) Rapamycin, but not resveratrol or simvastatin, extends life span of genetically heterogeneous mice, *J Gerontol A Biol Sci Med Sci*, **66**, 191-201.
- Miller, R.A., *et al.* (2014) Rapamycin-mediated lifespan increase in mice is dose and sex dependent and metabolically distinct from dietary restriction, *Aging Cell*, **13**, 468-477.
- Narbonne-Reveau, K., Charroux, B. and Royet, J. (2011) Lack of an antibacterial response defect in Drosophila Toll-9 mutant, *PLoS One*, **6**, e17470.
- Oliveira, R.P., *et al.* (2009) Condition-adapted stress and longevity gene regulation by Caenorhabditis elegans SKN-1/Nrf, *Aging Cell*, **8**, 524-541.
- Pan, D., *et al.* (2012) SnoN activates p53 directly to regulate aging and tumorigenesis, *Aging Cell*, **11**, 902-911.
- Partridge, L., *et al.* (2011) Ageing in Drosophila: The role of the insulin/Igf and TOR signalling network, *Exp Gerontol*, **46**, 376-381.
- Pickett, C.L. and Kornfeld, K. (2013) Age-related degeneration of the egg-laying system promotes matricidal hatching in Caenorhabditis elegans, *Aging Cell*, **12**, 544-553.

- Piper, M.D., *et al.* (2010) Water-independent effects of dietary restriction in Drosophila, *Proc Natl Acad Sci U S A*, **107**, E54-56; author reply E57.
- Rana, A., *et al.* (2010) Pantethine rescues a Drosophila model for pantothenate kinase-associated neurodegeneration, *Proc Natl Acad Sci U S A*, **107**, 6988-6993.
- Rawson, J.M., *et al.* (2012) Effects of diet on synaptic vesicle release in dynactin complex mutants: a mechanism for improved vitality during motor disease, *Aging Cell*, **11**, 418-427.
- Saiki, R., *et al.* (2008) Altered bacterial metabolism, not coenzyme Q content, is responsible for the lifespan extension in Caenorhabditis elegans fed an Escherichia coli diet lacking coenzyme Q, *Aging Cell*, **7**, 291-304.
- Selman, C., *et al.* (2008) Evidence for lifespan extension and delayed age-related biomarkers in insulin receptor substrate 1 null mice, *FASEB J*, **22**, 807-818.
- Selman, C., *et al.* (2008) Comment on "Brain IRS2 signaling coordinates life span and nutrient homeostasis", *Science*, **320**, 1012; author reply 1012-1012; author reply 1012.
- Selman, C., Partridge, L. and Withers, D.J. (2011) Replication of extended lifespan phenotype in mice with deletion of insulin receptor substrate 1, *PLoS One*, **6**, e16144.
- Selman, C., *et al.* (2009) Ribosomal protein S6 kinase 1 signaling regulates mammalian life span, *Science*, **326**, 140-144.
- Slack, C., *et al.* (2011) dFOXO-independent effects of reduced insulin-like signaling in Drosophila, *Aging Cell*, **10**, 735-748.
- Slack, C., *et al.* (2010) Regulation of lifespan, metabolism, and stress responses by the Drosophila SH2B protein, Lnk, *PLoS Genet*, **6**, e1000881-e1000881.
- Sofola, O., *et al.* (2010) Inhibition of GSK-3 ameliorates Abeta pathology in an adult-onset Drosophila model of Alzheimer's disease, *PLoS Genet*, **6**, e1001087.
- Spanier, B., *et al.* (2010) Altered signalling from germline to intestine pushes daf-2;pept-1 Caenorhabditis elegans into extreme longevity, *Aging Cell*, **9**, 636-646.
- Strong, R., *et al.* (2013) Evaluation of resveratrol, green tea extract, curcumin, oxaloacetic acid, and medium-chain triglyceride oil on life span of genetically heterogeneous mice, *J Gerontol A Biol Sci Med Sci*, **68**, 6-16.
- Strong, R., *et al.* (2008) Nordihydroguaiaretic acid and aspirin increase lifespan of genetically heterogeneous male mice, *Aging Cell*, **7**, 641-650.
- Toivonen, J.M., *et al.* (2007) No influence of Indy on lifespan in Drosophila after correction for genetic and cytoplasmic background effects, *PLoS Genet*, **3**, e95.
- Wang, C.T., *et al.* (2012) Reduced neuronal expression of ribose-5-phosphate isomerase enhances tolerance to oxidative stress, extends lifespan, and attenuates polyglutamine toxicity in Drosophila, *Aging Cell*, **11**, 93-103.
- Ye, X., *et al.* (2014) A pharmacological network for lifespan extension in Caenorhabditis elegans, *Aging Cell*, **13**, 206-215.
